# Advancing Precision Medicine in Myocarditis: Current Status and Future Perspectives in Endomyocardial Biopsy-Based Diagnostics and Therapeutic Approaches

**DOI:** 10.3390/jcm12155050

**Published:** 2023-07-31

**Authors:** Christian Baumeier, Dominik Harms, Ganna Aleshcheva, Ulrich Gross, Felicitas Escher, Heinz-Peter Schultheiss

**Affiliations:** 1 Institute of Cardiac Diagnostics and Therapy, IKDT GmbH, 12203 Berlin, Germany; dominik.harms@ikdt.de (D.H.); ganna.aleshcheva@ikdt.de (G.A.); ugross@zedat.fu-berlin.de (U.G.); heinz-peter.schultheiss@ikdt.de (H.-P.S.); 2 Department of Infectious Diseases, Robert Koch Institute, 13353 Berlin, Germany; 3 Department of Cardiology, Angiology and Intensive Care Medicine, Deutsches Herzzentrum der Charité, Campus Virchow Klinikum, 13353 Berlin, Germany; felicitas.escher@dhzc-charite.de; 4 German Centre for Cardiovascular Research (DZHK), Partner Site Berlin, 10785 Berlin, Germany

**Keywords:** endomyocardial biopsy, myocarditis, viral myocarditis, inflammatory cardiomyopathy, precision medicine, antiviral therapy, miRNA, gene expression profile, next-generation sequencing

## Abstract

The diagnosis and specific and causal treatment of myocarditis and inflammatory cardiomyopathy remain a major clinical challenge. Despite the rapid development of new imaging techniques, endomyocardial biopsies remain the gold standard for accurate diagnosis of inflammatory myocardial disease. With the introduction and continued development of immunohistochemical inflammation diagnostics in combination with viral nucleic acid testing, myocarditis diagnostics have improved significantly since their introduction. Together with new technologies such as miRNA and gene expression profiling, quantification of specific immune cell markers, and determination of viral activity, diagnostic accuracy and patient prognosis will continue to improve in the future. In this review, we summarize the current knowledge on the pathogenesis and diagnosis of myocarditis and inflammatory cardiomyopathies and highlight future perspectives for more in-depth and specialized biopsy diagnostics and precision, personalized medicine approaches.

## 1. Introduction

Myocarditis, as defined by the World Health Organization, is an inflammation of the myocardium [1,2,3]. In contrast, inflammatory cardiomyopathy (DCMi) is an inflammatory myocardial disease that is associated with systolic and/or diastolic dysfunction as well as cardiac remodeling [4]. Myocarditis may occur in the acute, subacute, or chronic phases with focal or diffuse involvement of the myocardium, and its prevalence has been reported as 10.2 to 105.6 per 100,000 individuals, with an annual occurrence of about 1.8 million cases worldwide [5].

In about half of the patients—mainly young people between 20 and 50 years of age without other significant health problems [6]—acute myocarditis resolves spontaneously without chronic cardiac dysfunction or serious pathophysiological sequelae. In the remaining cases, adverse outcomes such as life-threatening arrhythmias, requirement for a heart transplantation, and cardiac death may occur [7]. One of the major long-term complications of myocarditis, affecting approximately 12–25% of myocarditis patients [7], is the development of dilated cardiomyopathy (DCM) leading to chronic heart failure [8]. Accordingly, up to 10% of initially unexplained “idiopathic” cases of cardiomyopathy are thought to be caused by undiagnosed myocarditis [9].

Endomyocardial biopsies (EMBs) remain the widely accepted gold standard for the diagnosis of myocarditis [10]. For a specific EMB-based diagnosis and subsequent therapy recommendation, (immuno)histological examination in combination with PCR detection of cardiotropic viruses is required [4,11]. Because the primary cause of myocarditis is usually viral infection or autoimmunity [1,4], and treatment approaches differ between virus-negative patients and patients with evidence of active viral infection, comprehensive differential diagnoses on the basis of EMB analysis is essential for the management of myocarditis patients [12,13]. In order to improve the sensitivity of EMB in detecting myocarditis, it is crucial to obtain tissue samples from multiple ventricular regions, as myocarditis can manifest as focal, patchy, or diffuse processes [14].

A complementary approach for the diagnosis of myocarditis is cardiac magnetic resonance imaging (CMR) [6]. It is based on the Lake Louise Criteria (LLC) including T1 and T2 mapping and has been shown to assess severe inflammation and fibrosis [15]. However, the diagnostic power of CMR is limited because CMR is unable to detect the underlying etiology of myocarditis, particularly the presence of viral infections and transcriptional activity and the quality and intensity of inflammation cannot be accurately detected [11,16,17]. Therefore, no therapeutic decision can be made based on imaging alone [18]. Thus, initiation of immunosuppressive therapy should occur only after exclusion of active infection by EMB diagnostics [4]. Alternative imaging modalities such as spectral cardiac computed tomography and positron emission tomography (PET)-CMR to quantify and monitor myocardial inflammation may be useful in the diagnosis of acute myocarditis, particularly in cases of cardiac sarcoidosis [19]. Despite the obvious limitations, the utility of non-invasive imaging techniques in the diagnosis of myocarditis is undisputed, as reviewed elsewhere [20].

While histological examination of EMBs was originally used to identify inflammatory infiltrates associated with necrosis and/or degeneration of adjacent myocytes (Dallas criteria), diagnostic EMB criteria have been expanded to include additional factors such as immunohistochemical characterization and quantification of immune cells, virus-specific PCR, miRNA and cardiac antibody evaluation, and gene profiling. Accordingly, the most recent guidelines recommend EMB diagnostics for all individuals with suspected myocarditis as a basis for a causal and personalized treatment [4]. 

Prognosis can be determined based on EMB results, which provide insights into the underlying etiology and the type and intensity of inflammation [21]. Interestingly, fulminant myocarditis tends to have a more favorable outcome compared to the chronic form of the disease [22]. Unfortunately, as EMB remains an invasive procedure that is not always performed, the prognosis for many patients remains uncertain. However, the rapid development of (immuno)histological and virological methods, as well as long-term follow-up of myocarditis patients, has led to more accurate EMB diagnoses, which is highlighted in this review. In addition, current developments and future perspectives in EMB diagnostics will be discussed to highlight the advantages of this standard procedure compared to widely used imaging techniques, which remain inadequate in providing comprehensive characterization of the specific causative mechanisms of the disease as the basis for a therapeutic decision.

## 2. Histological and Immunohistochemical Inflammation Diagnostics

### 2.1. Histological EMB Evaluation—The Dallas Criteria

In addition to pathogen detection, histological studies of EMBs are the basis for appropriate diagnosis of acute myocarditis and chronic DCMi in clinical practice. In 1986, the histopathological Dallas criteria were introduced for the diagnosis of myocarditis in EMB specimen [23]. They include the detection of inflammatory infiltrates (which may be focal or diffuse) and non-ischemic myocyte necrosis or damage. However, because myocarditis often presents as a patchy inflammatory infiltrate and chronic forms of cardiac inflammation do not present with cardiomyocyte necrosis, there is a risk of underestimation based on these histological criteria alone. Already in the 1990s, retrospective analyses revealed that the diagnosis of myocarditis based on the Dallas criteria is of limited prognostic value in the routine evaluation of heart failure patients [24]. To date, several studies have confirmed that the Dallas criteria lack sensitivity and specificity as well as prognostic significance due to problems of sampling error and wide scope for interpretation [25,26,27,28,29]. Nevertheless, the Dallas criteria are still used today, as the histological detection of myocytolysis is essential for the diagnosis of acute myocarditis.

### 2.2. Immunohistochemical EMB Analysis

**Figure 1:**
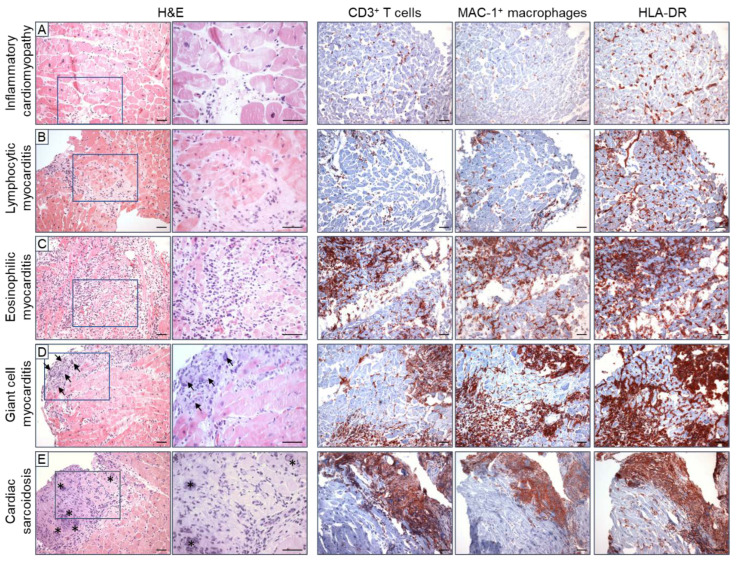
Various forms of acute myocarditis and inflammatory cardiomyopathy demonstrated by histological and immunohistochemical staining using endomyocardial biopsies. (**A**) Inflammatory cardiomyopathies are characterized by diffuse, low-grade infiltration of CD3^+^ T cells and MAC-1^+^ macrophages, as well as HLA-DR activation. (**B**) Lymphocytic myocarditis is also characterized by diffuse infiltration of mainly lymphocytic cells and HLA-DR activation, in combination with myocytolysis. (**C**) In eosinophilic myocarditis, the main inflammatory cells are eosinophilic granulocytes and macrophages. (**D**) Giant cell myocarditis is characterized by patchy infiltration of mononuclear and lymphocytic cells, HLA-DR activation, myocytolysis, and eosinophils in the presence of giant cells (arrows). (**E**) Cardiac sarcoidosis is also characterized by evidence of granulomas (*) and patchy immune cell infiltrates plus HLA-DR activation, but without myocytolysis. Magnification: 200× or 400×, scale bar: 50 µm.

As early as 1996, it was postulated that immunohistochemical analysis of cardiac biopsies increases the sensitivity of EMBs and might be essential for the diagnosis of myocarditis and DCMi [30]. During myocarditis infiltration of diverse immune cells including neutrophils, monocytes, macrophages, eosinophils, mast cells, natural killer cells, as well as T and B lymphocytes into the myocardium is observed. In comparison, DCMi tends to be dominated by T lymphocytes and macrophages, whereas B cells and NK cells are often not elevated [31]. Moreover, immunohistochemical diagnosis of myocarditis must be divided into acute and chronic forms, which differ according to the type of inflammatory cells and their distribution [32]. Acute forms are divided into lymphocytic, eosinophilic, giant cell, and granulomatous (cardiac sarcoidosis) myocarditis (Figure 1), with the latter three being rare and lymphocytic myocarditis accounting for 70% of the cases [33]. In contrast, chronic forms often have a lower number of inflammatory cells and differ in their immune cell composition [21]. Accordingly, detection of immune cell-specific surface antigens as well as other components of an active immune response by immunohistochemical methods has been established to improve the diagnostic and prognostic value of EMB. As a result, immunohistochemical examination of EMBs has become the gold standard for the diagnosis of myocarditis and DCMi, as suggested by the 2013 guidelines of the European Society of Cardiology Working Group [4]. Thus, a cut-off value of ≥14 leukocytes per mm^2^ is recommended, including up to 4 monocytes per mm^2^, with the presence of ≥7 CD3^+^ T lymphocytes per mm^2^. However, recent data show that limiting the criteria to CD3^+^ T cells and macrophages underestimates inflammation, as other cell types and adhesion molecules are also prognostically relevant. A recent meta-analysis on immunohistochemical detection of DCMi, which included 61 publications with a total of 10,491 patients, showed that diverse immunohistochemical criteria with different thresholds are applied in the diagnosis of myocardial inflammation [34]. Therefore, standardized immunohistochemical measurement of differentiated inflammatory markers is important for accurate EMB-based diagnosis of myocarditis/DCMi in the future.

### 2.3. Prognostic Relevance of Immunohistochemical Markers

The prognostic relevance of the diagnostic criteria has been partially demonstrated in smaller retrospective studies. Here, most of the studies focus on CD3^+^ T cells as the major contributor to inflammation. In a study from Lithuania, elevated cardiac levels of CD3^+^ T cells and CD45R0^+^ memory T cells were found to predict poor long-term prognosis in DCM patients [35]. Cut-off values of ≥13 CD3^+^ and ≥11.5 CD45R0^+^ cells/mm^2^ were calculated to best separate good and poor prognosis. In agreement, a Japanese multicenter study (INDICATE) including 261 DCM patients demonstrated that myocardial CD3^+^ T lymphocytes are an independent prognostic risk stratification marker for heart failure [36]. The authors divided DCM patients into patients with low, intermediate, and high inflammation levels according to their initial CD3^+^ T cell levels, and showed that patients were best classified into three risk categories with cut-off values of 13 cells/mm^2^ and 24 cells/mm^2^, respectively. Consequently, the higher the infiltration of CD3^+^ T cells, the lower the 10-year survival rate (CD3-low: 83.4%; CD3-intermediate: 68.4%; and CD3-high: 21.1%; Log-rank *p* < 0.001). Interestingly, the prognostic significance of CD68^+^ macrophages could not be demonstrated in this study, as the majority of patients (96%) exceeded the upper limit of ≥4 CD68^+^ cells/mm^2^ according to ESC guidelines. This suggests that the cut-off value for monocytes/macrophages proposed in the 2013 guidelines is not adequate. Another Japanese study involving 182 consecutive DCM patients, confirmed that elevated myocardial immune activation was associated with poor long-term outcome [37]. Detection of ≥14 CD3^+^ T lymphocytes/mm^2^ or ≥32 CD68^+^ macrophages/mm^2^ was associated with an increased risk of cardiovascular death or need for heart transplantation.

In line with the response to immunosuppressive therapy in patients with virus-negative chronic DCMi, Chimenti et al. demonstrated the prognostic significance of Toll-like receptor 4 (TLR-4) [38]. Here, expression of myocardial TLR-4 was associated with the LVEF after 6 months of immunosuppression. Furthermore, perforin was shown to have prognostic relevance. Two studies showed that the number of perforin-positive cytotoxic T cells in the myocardium predicts the outcome of DCMi patients [39,40].

**Figure 2:**
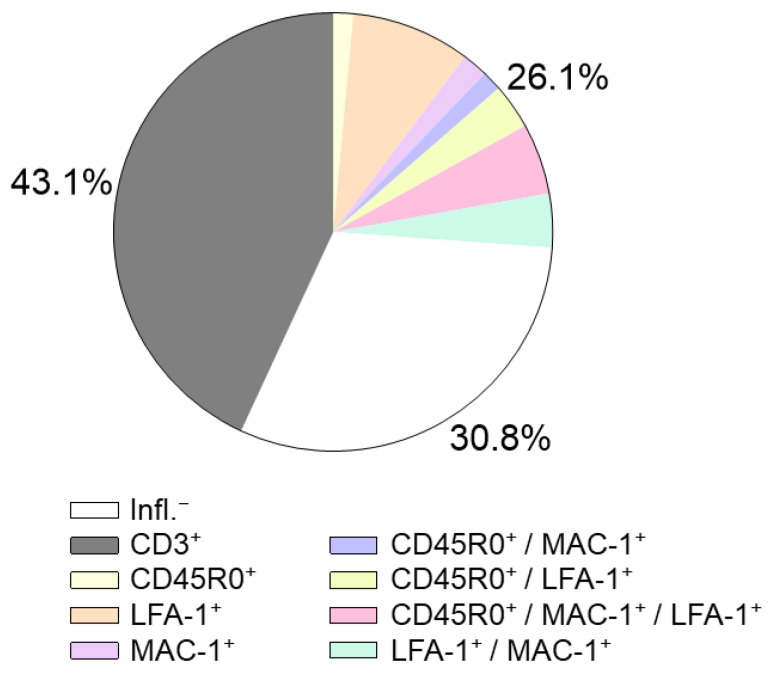
Differentiated evaluation of immunohistochemical markers in EMBs refines the diagnosis of intramyocardial inflammation. In a patient cohort of 450 patients with unexplained heart failure, 30.8% were characterized as inflammation negative (white) by immunohistochemistry, 43% of the patients showed elevated CD3 levels (grey), while the remaining 26% showed CD3 levels below the cut-off value (colored). Consideration of additional markers such as CD45R0, MAC-1, and LFA-1 provides evidence of inflammation in these patients despite normal CD3 levels (own data). These data indicate that an expansion of immunohistochemical parameters is urgently needed.

Our own data from more than 450 virus-negative heart failure patients showed that consideration of further inflammatory markers such as CD45R0 and LFA-1 in addition to CD3 T-cell and CD68 macrophage markers may improve detection of inflammatory infiltrates (Figure 2). In not considering these markers, 26% of patients with inflammatory processes would be missed. 

### 2.4. Further Immunohistochemical Markers

#### 2.4.1. Lymphocytes

The detection of lymphocytic infiltrates is a prerequisite for the diagnosis of myocarditis and DCMi, although the use of different immunohistochemical markers for the detection of inflammatory infiltrates complicates a standardized diagnosis of myocarditis/DCMi. The leukocyte common antigen CD45 is suggested to be more suitable for the diagnosis of lymphocytic myocarditis [41]. However, despite a threefold higher sensitivity of CD45^+^ compared to CD3^+^ in a preclinical study, CD45 has not been regularly used for the diagnosis of myocarditis. Further markers for T lymphocytes (CD2^+^) [42], B lymphocytes (CD20^+^) [43], helper T cells (CD4^+^) [44], cytotoxic T cells (CD8^+^ [45], perforin^+^ [39]) have been tested, with varying prognostic relevance.

For instance, the cytotoxic T cell marker perforin was shown to be an important predictor of LVEF deterioration [39] and mortality [40] in patients with DCMi. Other markers of cytotoxic lymphocytes, such as granzyme B, involved in post-ischemic cardiac remodeling have also been proposed as diagnostic markers for the detection and differentiation of cardiac inflammation [46].

Differentiation of T cell responses in EMBs could also improve prognostic assessment and specific treatment decisions. Autoreactive CD4^+^ T cells are associated with autoimmunity [47,48], while cytotoxic CD8^+^ T cells play a role in fulminant myocarditis [49] and antiviral defense [45,50]. Since CD4^+^ T cells are the major cause of autoimmune myocarditis [51,52] and are involved in the transition from hypertrophy to heart failure [53], determination of CD4^+^ cell numbers may facilitate the diagnosis of myocardial damage caused by autoimmunity, as observed in the myocarditis in response to COVID-19 vaccination [54]. In contrast, specific inhibition of CD8^+^ T cells could be used for the treatment of acute myocarditis, as already shown in the setting of acute myocardial infarction [46]. Therefore, differentiating between acute and chronic forms of myocarditis based on the CD4+/CD8+ ratio may be a viable option. With regard to autoimmunity, markers for T helper 17 (T_H_17) cells and hypoxia-inducible factor-1 alpha (HIF-1a) could be useful [55]. Since persistent heart failure is associated with high number of IL-17-producing T cells [56], detection of T_H_17 cells in EMBs could be of high prognostic importance. IL-17 is also a promising target with regard to therapeutic approaches.

#### 2.4.2. Monocytes and Macrophages

Cells of the mononuclear phagocyte system, primarily monocytes and macrophages, are involved in the regulation of cardiac inflammation in human myocarditis [57]. Here, they are responsible for tissue repair, regeneration, and fibrosis [58]. Unlike in infarct-induced damage to the myocardium, little is known about the role of macrophage subtypes in myocarditis [59]. Polarization of bone marrow-derived monocytes to macrophages takes place in the myocardium and is highly dependent on the cardiac microenvironment including cytokine and chemokine levels, the inflammatory milieu, as well as fibroblast presence [60]. It is hypothesized that M1-like macrophages exacerbate inflammation and lead to heart failure and that M2-like macrophages suppress inflammation and contribute to tissue repair. In an EMB-based study on 176 patients with DCM or DCMi, we showed that the polarization of cardiac macrophages into an M1 or M2 fate depends on the degree of lymphocytic inflammation and the expression of cardioprotective plasminogen activator inhibitor type-1 (PAI-1), a well-known contributor to tissue fibrosis [61].

The prognostic relevance of macrophage subtypes In cardiac inflammatory disease is still unknown and needs to be analyzed in future studies. Importantly, confocal detection of M1-specific (e.g., CD14^+^, CD86^+^) and M2-specific (CD163^+^, CD16^+^) surface antigens, in combination with the pan-macrophage marker CD68^+^, is highly recommended for the classification of polarized macrophages [60,61,62].

#### 2.4.3. Cell Adhesion Molecules and the Human Leukocyte Antigen System

The determination of cell adhesion molecules (CAMs) and human leukocyte antigen (HLA) expression in EMBs can provide important information about the cause of inflammation. The presence of immune cell infiltrates in combination with abnormal expression of HLA class II and/or CAMs in virus-negative EMB samples is indicative of an autoimmunological inflammatory response [63,64]. As CAM activation is observed in 67% of DCM patients and its expression strongly correlates with the immunohistochemical diagnosis of DCMi, evaluation of CAMs might be of diagnostic relevance in DCM and DCMi patients [65]. For example, vascular cell adhesion molecule 1 (VCAM-1) expression in the myocardium is closely associated with immune cell infiltration and increased risk of heart failure [66]. Since VCAM-1 is discussed as biomarker for the prediction of cardiovascular disease [67], routine determination of VCAM-1 levels in EMBs could be used for risk-stratification in myocarditis patients. Intercellular adhesion molecule-1 (ICAM-1), on the other hand, is expressed on cardiac myocytes of patients with myocarditis and is associated with chronic inflammation [68]. A study on mice with experimentally induced heart failure demonstrated that ICAM-1 regulates cardiac pathological remodeling by mediating left ventricular (LV) leukocyte infiltration, fibrosis, and dysfunction of the heart [69]. Moreover, ICAM-1 has been proposed as a therapeutic target in heart failure patients [70]. Finally, the diagnostic value of HLA typing has been widely discussed [71]. HLA antigens have been reported to associate with DCMi [2] and a genome-wide association study identified HLA as a risk locus for DCM, consistent with an autoimmunological etiology [72].

## 3. Virus Diagnostics

Next to idiopathic and autoimmune pathogeneses, viral infection is one of the most common causes of myocarditis and chronic heart failure. Although coxsackieviruses were the viruses that were most commonly associated with myocarditis in the 20th century, a shift in detected cardiotropic viruses has been observed in recent decades, concurrent with the switch from traditional serology-based methods to the more sensitive nucleic acid tests such as in situ hybridization, and later polymerase chain reaction (PCR) [73,74]. Several studies have reported an increased incidence of parvovirus B19 and herpes viruses, such as human herpes virus 6, in endomyocardial biopsies [75,76,77]. Furthermore, new data show that active viral transcriptional activity plays a determining role in patient outcome, thereby necessitating looking beyond the mere detection of inflammation and viral genomes to evaluate the prognosis as the basis of establishing the best course of treatment for patients [78]. While the various cardiotropic viruses differ in their initial pathogenic mechanisms within the heart, they share the same principle clinical outcomes following the acute replicative stages. Either the virus is quickly cleared from the myocardium by the infiltrating immune cells and inflammation subsequently abates, leading to a full recovery with usually minimal or no residual injury. Or the infection persists, either with or without inflammation, caused by repeated exposure to viral antigens. A final outcome is viral clearance without cessation of immune activation, leading to autoimmune inflammation and continuing damage to the heart. In the latter cases, a chronic phase defined by the associated cardiac remodeling ultimately leads to a clinical picture of DCM. However, apart from published prognostic data on enterovirus or active parvovirus B19 infection [78,79], the impact of cardiac viral persistence on patient outcome remains uncertain. An overview of the most commonly detected cardiotropic viruses, their cell tropism, clinical manifestations, and pathophysiology, as well as appropriate therapeutic approaches is provided in Table 1.

### 3.1. Enteroviruses and Adenoviruses

In the latter half of the 20th century, enteroviruses (EV), specifically the coxsackievirus A and B groups, were considered to be the most common etiological agents of viral myocarditis [117]. Both immunohistochemical analysis of viral VP1 protein and detection of genomic and antigenomic viral RNA have proven that EVs indeed replicate within cardiomyocytes [73,118,119]. Research has shown that viral negative-strand genomes, as indicators of EV replication, are present in the myocardium of patients with LV dysfunction and suspected myocarditis [120]. Viral replication and translation of the viral protease 2A causes myocyte necrosis and leads to fibrosis and cardiomyopathy mainly due to the cleavage of dystrophin in the chronic phase [93,121]. Clinically, active EV infection contributes to sudden death in other forms of heart disease, such as myocardial infarction, and viral replication is further associated with decreased LV function [122]. Enteroviral persistence in the myocardium was reported in up to 50% of EMB-proven cases of infection, with further decreases in LV function [79]. Viral mutations and their functional consequences appear to favor persistent infection and development of DCM [94]. Correspondingly, patient survival was shown to significantly decrease over time in persisting EV-related DCMi, while spontaneous clearance was associated with more favorable outcomes [123]. 

Like EVs, the dsDNA adenoviruses (AdV) were considered a major cause of non-ischemic heart disease alongside EVs as reported by EMBs or autopsy-based studies in the 1990s and early 2000s [124,125,126]. However, detection of other cardiotropic viruses began to be more frequent thereafter, with recent studies suggesting that both EVs and AdV no longer play a dominant role in viral myocarditis [75,76,77,127,128]. Whether this shift truly reflects an epidemiological change or merely a trend of better detection of previously underreported viral infections has yet to be determined. In the meantime, routine testing for entero- and adenoviruses in patient EMBs remain necessary for comprehensive cardiac diagnostics.

### 3.2. Parvovirus B19

**Figure 3:**
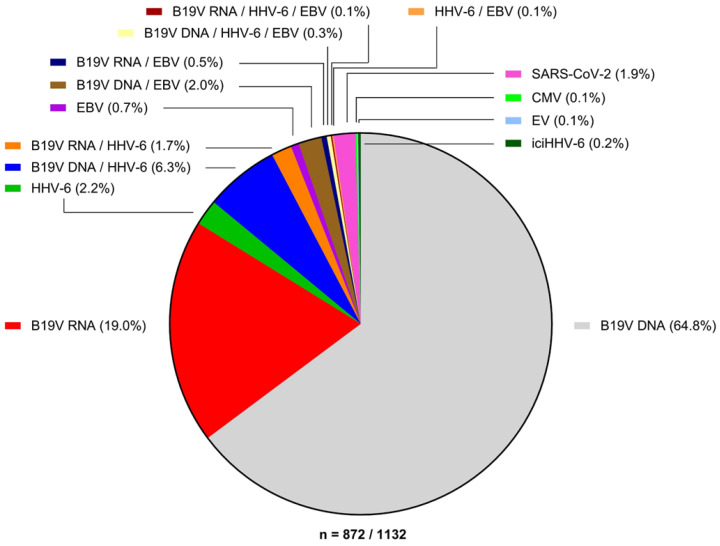
Incidence of viral infections in endomyocardial biopsies of *n* = 872 virus-positive patients. A consecutive cohort of EMBs from 1132 patients with unclear heart failure was tested for presence of cardiotropic viruses via PCR. A total of 872 samples were virus positive (77%), of which B19V viral DNA alone (B19V DNA, 64.8%) or in combination with viral transcripts (B19V RNA, 21.3%) were the main contributors. SARS-CoV-2 constituted 1.9% of virus-positive cases. EV, HHV-6, EBV, and CMV, as well as co-infections therewith constitutes the remaining bulk of cases (14.1%). Detection of iciHHV-6 in heart failure patients was rare (0.2%).

A comprehensive study in 2005 found that 67.4% of EMBs from patients with “idiopathic” dilated cardiomyopathy in fact harbored viral genomes [75]. Astonishingly, while evidence of EV infection was comparatively low (9.4%), a human pathogenic ssDNA erythrovirus, parvovirus B19 (B19V), was detected in more than half (51.4%) of biopsies [75]. Novel data from 2021 on 1132 consecutive EMBs show virus positivity in 77% of the cases. Among all virus-positive patients, B19V incidence was 95% (Figure 3). Thus, B19V is the most frequently detected cardiotropic virus in EMBs of heart failure patients. It primarily infects erythroblasts and causes erythema infectiosum or fifth disease in early childhood, or otherwise causes hydrops fetalis during pregnancy, transient aplastic crisis in those with hemolytic disorders, and pure red cell aplasia in kidney transplant patients. In the adult myocardium, however, B19V is found in vascular endothelial cells, likely after migration of infected bone marrow-derived endothelial progenitors to sites of vessel repair [129]. This endothelial infection leads to endothelial disruption and dysfunction both within systemic as well as myocardial vessels, with subsequent myocyte necrosis secondary to impaired microcirculation [97,101,130]. Furthermore, the increased incidence of coronary and microvascular spasms is associated with B19V myocarditis [96]. Infection of circulating angiogenic cells by B19V contributes to impaired endothelial regeneration, enhancing cardiac ischemia in B19V-associated cardiomyopathy, with the data additionally showing viral transcriptional activity in half of the presented cases [131,132]. Previously, a limit of 500 viral genome copies/µg DNA was suggested to allow the differentiation between a possible bystander and actively virulent role of B19V [133]. This assumption has been called into question in recent years, with survival data showing no significant differences based on viral genome load, but appear to be dependent on other viral and inflammatory factors [78]. Concordantly, while viral clearance rates appear to be low, infections are only pathophysiologically relevant when accompanied by either immunohistochemically detected chronic inflammation, viral RNA transcripts, or both [78,79]. Indeed, the detection of viral NS1 and VP1/VP2 RNAs has been found to be a crucial prognostic parameter, as viral transcriptional activity is correlated with adverse long-term prognosis in non-ischemic heart failure patients [78,134]. At the same time, reduction or elimination of B19V transcripts in the heart by antiviral therapy has been shown to improve LV function [105,108]. Approximately 15% of patients positive for B19V DNA in EMBs have detectable viral transcriptional activity [104], whereas more recent data show a rather increased incidence of 21.3% (Figure 3). The immunohistochemical detection of B19V capsid proteins VP1 and VP2 has also been reported in EMBs of DCM patients, strengthening the observation that limited yet pathogenic viral transcriptional and translational programs hold high relevance in B19V-associated heart disease [135]. Furthermore, dysregulation of inflammatory and mitochondrial gene expression was reported in patients with detected active viral replication, returning to levels of healthy controls after spontaneous viral clearance [104]. B19V transcription thus directly damages cellular energy metabolism and enforces an inflammatory response, thereby contributing to the pathogenesis of myocarditis and DCMi [104]. However, it appears that B19V not only alters the cellular environment during active transcription, but also causes ultrastructural changes and damage to myocardial mitochondria in the absence of viral transcription and myocarditis, thereby challenging the notion that the presence of B19V genomes alone is clinically non-relevant [136]. Reports over the years have reinforced B19V’s role in inflammatory heart disease and DCM. B19V infection is significantly correlated with DCMi, and co-infections with other cardiotropic viruses are common [137]. Human herpes virus 6 (HHV-6) may act as a transactivator for B19V viral transcription via its U94 protein, potentially exacerbating infections [137]. Indeed, co-infections with HHV-6 are common (Figure 3) [75,77,103,137,138,139,140]. Interactions with other potentially cardiotropic viruses, such as influenza A, have been shown to lead to detrimental outcomes [141]. Viral latency and active infection may alternate in individuals, accelerating LV dysfunction and development of DCM [142]. B19V is commonly found in EMBs of patients with DCM, whereas little to no correlation was found with blood virus titers, and no detection can be achieved with imaging, underlining the need for a comprehensive biopsy-driven diagnostic workup in cases of DCM of unclear etiology to allow for sufficient patient characterization [143]. Finally, the presence of B19V in the pediatric heart likely increases the risk of graft loss [103]. Although present in three-quarters of patient EMBs, the role of the mere presence of B19V viral genomes is still under debate. However, viral re-activation and co-infection, as well as concomitant chronic inflammation and viral transcriptional activity, have been shown to correlate with development of DCM and poor prognosis. It is evident therefore, that assessment of B19V genomic and transcriptional presence is instrumental in clarifying the etiologies of myocarditis and inflammatory and dilated cardiomyopathies.

### 3.3. Herpesviridae

Several human herpes viruses have long been associated with viral myocarditis. The previously mentioned betaherpesvirus HHV-6 is subclassified into species 6A and 6B, that differ in their clinical presentation. HHV-6 has been found to be one of the most common viruses detected in EMBs of patients with myocarditis and DCM [75,76]. Explanted hearts from children with DCM had a high incidence of HHV-6B (43.7% and 8.3% in two different studies), and possible re-activation of the virus in heart transplant patients was observed via detection in EMBs [103,144]. A South African study found evidence of HHV-6 in 2/50 EMBs of patients with acute myocarditis, behind Epstein–Barr virus (6/50) and B19V (37/50) [77]. In another study, 26% of patients with coronary artery spasms had detectable HHV-6 in their EMBs and acute myocarditis [96]. Our own data revealed an incidence of 10.9% of all virus-positive EMBs from patients with heart failure (Figure 3). HHV-6B appears to be primarily associated with cardiotropism, with viral persistence in EMBs correlating with reduced LV function [145]. Furthermore, HHV-6 can stably integrate into the telomere region of human chromosomes during infection. If integration occurs in germline cells, offspring may carry the integrated viral genome in all nucleated cells, a condition dubbed inherited chromosomally integrated HHV-6 (iciHHV-6). Roughly 1% of the human population harbors iciHHV-6. In a large study cohort, 1.1% of EMBs from patients with heart failure had detectable iciHHV-6 titers, with parallel detection of viral RNA transcripts and glycoproteins, indicating re-activation [98]. Six of these patients were successfully treated with ganciclovir [98]. Finally, even in cases of iciHHV-6 in cardiac patients, evidence exists that superinfections with distinct HHV-6 strains are possible [99]. These studies and data paint HHV-6 as an emerging and highly relevant cardiotropic virus in both de novo infections, re-activations, and inherited forms, requiring comprehensive molecular diagnostic workup if viral involvement in heart disease is suspected. Herpes viruses in general should be considered during the diagnostic procedure in immunocompromised patients with cardiac symptoms. In immunocompromised individuals, such as transplant recipients, and those with latent HHV-6, Epstein–Barr virus (EBV), and human cytomegalovirus (HCMV) infections can re-activate and contribute to various cardiovascular diseases, including myocarditis, transplant vasculopathy, hypertension, restenosis, and atherosclerosis.

EBV-related myocarditis has been reported in children receiving heart transplants [84]. Acute EBV-related myocarditis is rare but can pose life-threatening complications in immunocompetent patients, possibly due to reactivation of a latent EBV infection [146,147,148]. Typical clinical manifestations of EBV infection may be absent even in fatal cases, and lymphocytic infiltration into the myocardium with myocyte necrosis has been reported [149]. This immune-mediated damage may be led by CD8^+^ T cells [81]. Reported cases, although rare, have generally been severe, even requiring heart transplantation, although this may reflect an underreporting of the subclinical presence of EBV in the heart [150]. Electron microscopy studies have revealed the presence of EBV antigens in cardiomyocytes and intramural vessels of patients with myocarditis and DCMi, despite the virus’ general tropism for B lymphocytes [82,83]. Although considered a rare cardiotropic pathogen, the potential for re-activation of latent EBV, and its possible tropism for cardiomyocytes, makes it necessary to routinely test for the presence of this virus in EMBs. 

Human CMV, or human betaherpesvirus 5 (HHV-5), like EBV, rarely causes myocarditis in immunocompetent individuals. Suspected cases of CMV-related myocarditis are usually based on serology and imaging, and not EMBs [92,151,152,153,154]. Intriguingly, however, a Finnish retrospective study found a high prevalence of CMV genomes in archived heart tissues of patients that suffered from fatal myocarditis [155]. Early studies showed that CMV-related myocarditis is not strictly a manifestation of a lymphotropic disease, but that CMV can actively infect cells of the heart vessels and the myocardium, including interstitial and endothelial cells in myocarditis cases [88,90,156]. Anti-herpes virus treatments have shown success in CMV-related myocarditis [92]. This, together with the fact that CMV re-activation is a common and serious event in transplant patients, should necessitate testing for CMV infection in suspected cases of viral cardiac involvement both in immunocompetent and immunocompromised persons.

### 3.4. SARS-CoV-2

During the COVID-19 pandemic, cardiac involvement of the novel severe acute respiratory syndrome coronavirus-2 (SARS-CoV-2) quickly became evident as reports of myocardial injury caused by severe lymphocytic and macrophage-dominated myocardial inflammation was reported [157,158,159]. Viral RNA can be detected in the myocardium of patients with suspected myocarditis linked to COVID-19 infections, even in the absence of positive nasopharyngeal swabs [112,159,160,161,162]. Infections also cause alterations in cardiac gene expression to promote antiviral and inflammatory responses [163]. In vitro studies have demonstrated that SARS-CoV-2 can infect cardiomyocytes via the angiotensin-converting enzyme 2 (ACE) and the serine protease TMPRSS2, which are highly expressed on these cells and additionally upregulated in patients with cardiomyopathies [115,164,165]. Myocardial injury is primary to direct cytotoxic damage of cardiomyocytes or direct (infection) and indirect (cytokine storm) damage of the microcirculation due to endothelial dysfunction [110,111,166,167]. The persistence of viral RNA in the myocardium post-COVID with associated reduced LV function and inflammation has been observed [114]. Autoimmune-mediated damage due to anti-heart antibodies and biopsy-proven persistent myocarditis and heart failure in “long COVID” is a clinically relevant outcome [113,114]. Although the height of the COVID-19 pandemic is over, SARS-CoV-2 is here to stay, and should be considered during EMB workups if a recent or current infection is indicated. Finally, increasing reports of myocarditis cases after COVID-19 vaccination have emerged, more often after administration of mRNA-based vaccines and in young male adults, with evidence of viral spike proteins present in the myocardium as confirmed by EMBs [54,168].

### 3.5. Other Cardiotropic Viruses

Several other DNA viruses have been sporadically associated with myocarditis/cardiomyopathy. In the herpes virus family, herpes simplex types 1 and 2 (HSV-1/2) are rare causes of heart disease [169,170,171]. Severe myocardial involvement of varicella zoster virus, another alphaherpesvirus, more commonly known to cause chicken pox and shingles, was described early in children [172,173]. However, immunocompetent adults may present with cardiac symptoms, often mimicking myocardial infarctions, as well as recurrence [174,175,176]. Finally, four clustered cases of HHV-7-associated with acute myocarditis and development of DCM have been reported [177]. Other RNA viruses, excluding enteroviruses, found globally and associated with myocarditis include influenza A and B viruses, measles and rubella viruses, respiratory syncytial virus, parainfluenza virus, hepatitis C and E viruses, and human immunodeficiency virus [141,178,179,180,181,182,183,184,185,186,187]. Finally, vector-borne infections caused by chikungunya, dengue, Zika, and West Nile viruses, have been associated with myocarditis [188,189,190,191,192,193,194,195,196]. These cases are rare, but should not be neglected, considering that 3.5 billion people live in areas where these viruses are endemic.

### 3.6. Incidence of Viral Myocarditis

A review of EMB samples with positive virus-specific PCR results from 1132 heart failure (Figure 3) patients from our lab identified latent B19V infection through the presence of only viral genomic DNA as the predominant finding (64.8%), followed by active B19V mono-infection with detectable transcriptional activity (19.0%), co-infections of latent or transcriptionally active B19V with HHV-6 (6.3% and 1.7%, respectively) or with EBV (2.0% and 0.5%, respectively), as well as mono-infection by HHV-6 (2.2%). SARS-CoV-2-positive EMBs made up 1.9% of the virus-positive samples. Detection of EV, EBV, or CMV alone (0.1%, 0.7%, and 0.11%, respectively) or combined with viruses other than B19V was rare. Similarly, the detection of iciHHV-6 was rather rare (0.2%).

## 4. Advances in EMB Diagnostics

### 4.1. Gene Expression Profiling

IGCM is recognized as a distinct clinical and pathological condition that exclusively affects the heart. This rapidly progressive disease is characterized by the necrosis of heart muscle cells and often leads to poor cardiac outcomes [197,198]. Consequently, early detection of IGCM is crucial in order to prevent or minimize progressive myocardial tissue damage, the need for eventual transplantation, or even cardiac death. Multiple multicenter studies have reported a five-year transplant-free survival rate of no more than 10% [199,200]. The presence of multinucleated giant cells (Figure 1), primarily found in lymphocytic infiltrates, myocytolytic tissue, and eosinophils, is a defining feature of IGCM [201,202]. However, due to their focal distribution within EMBs, conventional histologic evaluation often fails to detect giant cells accurately [203]. In fact, only 68% of initial EMBs yield a definitive diagnosis, necessitating repeat biopsies to confirm IGCM [204]. Consequently, due to underdiagnosis, the true incidence of IGCM may be higher than currently reported.

Recent studies have demonstrated that analyzing the gene expression profile of myocardial tissue reveals a distinct pattern indicative of IGCM, even in the absence of detectable giant cells on histological examination [205,206]. Specifically, we identified 5 genes (CPT1, CCL20, CCR5, CCR6, TLR8) out of 27 candidates that are associated with myocarditis and have high specificity for IGCM [207]. Conventional histologic examination alone would have missed 54.3% of IGCM cases without the use of this panel. Of note, patients diagnosed with IGCM based on this gene expression profile responded similarly to immunosuppressive therapy as patients diagnosed based on conventional histology alone [207]. These findings underscore the clinical utility of gene profiling in patients presenting with unexplained acute heart failure. The application of this novel gene profiling approach significantly improves the diagnostic rate in cases of clinical suspicion of myocarditis and unexplained acute cardiac decompensation and allows the detection of giant cells that might be missed by conventional histological examination. Consequently, this advancement has significant prognostic and therapeutic implications for patients. However, the application of this technique in clinical practice is limited to specialized laboratories, but can be established by simple molecular biology methods.

Over the past decade, gene expression profiling has been successfully integrated into clinical diagnoses. By employing gene profiling, the need for repeated EMBs could be eliminated, and immunosuppressive therapy could be initiated promptly, with treatment duration effectively monitored. Early diagnosis of IGCM and the initiation of immunosuppressive treatment are critical for optimal patient recovery, as they can prevent or minimize permanent myocardial damage, improve prognosis, and potentially obviate the need for heart transplantation.

### 4.2. miRNA Profiling

MicroRNAs (miRNAs) are a class of small ribonucleic acids (RNAs) consisting of 19 to 25 nucleotides [208]. Despite being non-coding molecules, they play a critical role in the regulation of gene expression [209]. Interestingly, a single miRNA molecule can target hundreds of different messenger RNAs (mRNAs) associated with essential cellular functions [210], and approximately one third of protein-coding gene regulation is influenced by miRNAs [211,212].

miRNAs have emerged as crucial players in the pathogenesis of various cardiovascular diseases, including myocardial infarction, arrhythmias, heart failure, and coronary heart disease [213]. As a result, there is great interest in investigating these small molecules in the context of myocarditis and heart failure [214]. miRNAs can be isolated not only from cells and tissues but also from body fluids like blood plasma or serum. Quantitative PCR (qPCR) is a widely used method for measuring miRNA levels from human material [215]. Additionally, it can be used to identify potential targets of the miRNAs under study, which may be valuable in elucidating various pathogenic pathways [208].

Numerous miRNA profiling studies have demonstrated significant dysregulation of different miRNAs in the blood and tissues of individuals with myocarditis. These miRNAs have the potential to differentiate myocarditis from other cardiac disorders, distinguish the stages of myocarditis, and provide prognostic information regarding the extent of cardiac damage and potential outcomes. 

Patients with viral myocarditis exhibited elevated levels of miR-133a and miR-155 in comparison to individuals with DCM [216]. These elevated levels showed a positive correlation with the inflammatory cell count. Furthermore, increased miR-133a was associated with reduced fibrosis and necrosis of myocytes, as well as improved LV function. Patients with elevated miR133a had a higher overall survival rate and a lower incidence of malignant arrhythmias. Additionally, they experienced fewer hospitalizations due to heart failure [216]. miR-1, miR-133a, miR-133b, miR-208a, and miR-208b were downregulated in the myocardial samples from patients with Chagas disease, which is mainly characterized by myocarditis, compared to healthy hearts from organ donors [217]. Moreover, miR-1, miR-133a, and miR-208b had significantly lower expression in the study group, compared to not only organ donors but also to DCM patients. Several target genes for these miRNAs were identified, indicating their involvement in cardiac hypertrophy, dysfunction, and normal cardiomyocyte proliferation [217]. Levels of miR-155 and miR-148a are elevated in the heart tissues of myocarditis patients compared to healthy individuals [218]. In contrast, miR-214 and miR-146b are downregulated in patients with viral myocarditis compared to healthy controls [219]. In our previous study, we focused on patients with active or latent B19V infection, which plays a role in both myocarditis and DCM, and identified 29 miRNAs with differential expression. The target genes of these altered miRNAs were associated with pathways related to cardiomyopathy, inflammatory responses, and mitochondrial energy metabolism [220].

Corsten et al. revealed the dysregulation of 107 different miRNAs in patients with viral myocarditis. The most upregulated miRNAs included miR-146b, miR-511, miR-155, miR-889, and miR-212, while the most downregulated miRNAs were miR-361, miR-106a, and miR-93 [221]. In patients with enteroviral cardiomyopathy, miR-135b, miR-155, miR-190, miR-422a, miR-489, miR-590, miR-601, and miR-1290 were only detected in EMBs of patients with persistent cardiac infection and clinical deterioration, and were found to be highly predictive [222]. Comparing patients with fulminant myocarditis to those with MI and healthy controls, Nie et al. found increased levels of miR-4281 in both the myocarditis and MI groups compared to healthy controls, while miR-4763-3p was significantly higher only in myocarditis patients [223]. Interestingly, the magnitude of the observed increase in miRNA levels was negatively correlated with the severity of fulminant myocarditis. Therefore, miR-4763-3p shows promise as a biomarker to differentiate between myocarditis and MI.

Interestingly, patients with fulminant myocarditis showed increased plasma levels of miR-29b and miR-125b. This upregulation was positively correlated with the extent of myocardial edema and negatively correlated with the LV function. However, miR-29b demonstrated higher sensitivity and specificity for diagnosing fulminant myocarditis compared with miR-125b [224]. In a study cohort with confirmed myocarditis, higher plasma levels of miR-155 and miR-206 were observed compared with a control group with DCM [225]. Another study demonstrated that patients with viral myocarditis had significantly increased levels of miR-21-5p and miR-1-3p in peripheral blood, compared to healthy volunteers [226].

Analysis of patients with fulminant myocarditis showed higher levels of miR-30a, miR-192, miR-146a, miR-155, and miR-320a compared to patients with non-fulminant myocarditis or healthy controls [227]. Importantly, combined analysis of miR-155 and miR-320a showed sufficient accuracy in distinguishing between fulminant and non-fulminant myocarditis. Notably, this miRNA panel demonstrated greater diagnostic value than the levels of C-reactive protein and cardiac troponins, even when analyzed together. As demonstrated by Fan et al., viral infection of the myocardium results in altered levels of five distinct miRNAs, of which miR-30a and miR-181d were related to enhanced IL-6 levels and thus contributed to an over-activated inflammatory response to viral infection [228]. Preliminary data from DCMi patients showed that specific miRNA profiles can be identified in EMBs from patients with active viral infection.

In our latest study, we evaluated the levels of various miRNAs in serum samples from patients with myocarditis and DCM, and healthy controls. Here, we found significant dysregulation of let-7f, miR-93, miR-197, miR-223, and miR-379 in patients with myocarditis. The expression of miR-93, miR-197, and miR-379 was increased compared with the other two groups, whereas let-7f and miR-223 levels were downregulated compared with healthy controls. The specificity of identifying myocarditis using these biomarkers in a single serum sample ranged from 93% to 95% [229].

In summary, numerous studies have been conducted to investigate the involvement of miRNAs in myocarditis. However, the majority of these studies have been preclinical, with relatively little emphasis on clinical research. Several clinical studies have demonstrated significant alterations in the levels of multiple miRNAs in serum (or plasma) and tissues of myocarditis patients. These observed dysregulations often correlate with the extent of heart damage, inflammation, and even cardiac death. These findings demonstrate the potential prognostic value of miRNAs in myocardial disease. Moreover, it is worth highlighting that the miRNA profiles in myocarditis patients differed from those observed in patients with other cardiac conditions, such as MI or non-inflammatory DCM. This distinction makes miRNAs promising biomarkers for the diagnosis of myocarditis and DCMi, as well as novel specific therapeutic options in the future.

### 4.3. Next-Generation Sequencing in Pathogen Detection

Detection of cardiotropic infections requires the use of EMBs, as serological methods suffer from low sensitivity and correlation, and imaging techniques cannot detect any viral causes. However, due to the limitation in obtaining tissue samples, the use of standard molecular techniques such as qPCR can often only test for a very limited number of pathogens before the material is exhausted. The shift in detected cardiotropic viruses in recent decades, as well as the SARS-CoV-2 pandemic, and the observation of different viruses based on geographical regions have demonstrated that the spectrum of pathogens involved in heart disease is constantly changing, evolving, and expanding. To ensure adequate diagnostic and prognostic performance in the future, novel methods to address this challenge must be developed. Clinical metagenomic approaches attempt to characterize the pathogen profile in a given clinical sample using massively parallel sequencing, also called next-generation sequencing (NGS). Different techniques are used, including direct shotgun sequencing of the extracted nucleic acids which gives the most accurate picture of the represented pathogens, but requires high input amounts and lacks sensitivity. Protocols using PCR-based (amplicon), single primer, or hybridization capture enrichment have all been described to increase metagenomic sensitivity [230,231,232]. The latter method has garnered much attention in recent years, as the parallel use of thousands of viral sequence-specific RNA baits to capture pathogen nucleic acids present in a sample theoretically enables the detection of all relevant viral species [233]. Specific software tools for the computation and design of such bait sets have been made available [234]. 

Most metagenomic studies focus on minimally invasive and abundant liquid biopsies, which constitute relatively simple sample matrices. These approaches may be useful for regular testing of blood viral loads in transplant patients, or even pathogen characterization in viral outbreak scenarios [235,236,237,238]. For solid tissue biopsies, the number of published protocols dwindles in comparison. Targeted viral metagenomics via hybridization capture and sequencing appear suited for solid biopsies. Capture of EBV genomes was demonstrated in a study on the genetic diversity of EBV in nasopharyngeal carcinomas [239]. A custom capture panel covering 535 human virus species was successfully applied to enrich polyoma virus sequences from brain tissue [240]. Finally, another study using a custom capture panel and metagenomic sequencing aimed to detect viruses in EMBs and explanted hearts of patients with fulminant or giant cell myocarditis [241]. Only sequences of endogenous retrovirus K were detected in myocardial tissue with this approach; however, the study suffered from low sample numbers (2 EMBs and 13 explanted hearts) [241]. Endogenous retrovirus K is also ubiquitous in the human genome. Nonetheless, a proof-of-principle for targeted metagenomics approaches for solid biopsy samples, including EMB, was given, which should be expanded upon in the future.

Several issues must be addressed for clinical metagenomics to make its way into routine diagnostics, however. The College of American Pathologist’s 2011 NGS Work Group developed an 18-point laboratory accreditation checklist for both wet lab and dry lab (bioinformatics) procedures for NGS [242]. These encompass documentation of the targeted enrichment procedure, quality control criteria to assess library preparation, enrichment, and sequencing run performance, internal validation including sensitivity and specificity testing, and validation of sequencing results by other methods (Sanger sequencing or PCR) [242]. Data storage and transfer policies, software version traceability, exception logs for deviations in standard bioinformatics pipelines, and procedures for the reporting of sequencing analysis results are part of the dry lab requirements. The European Society for Clinical Virology (ESCV) Network on Next-Generation Sequencing (ENNGS) has also established guidelines to accelerate the implementation of clinical metagenomics into diagnostic practice, with a special emphasis put on adequate internal process controls [243,244]. These requirements may be difficult for small diagnostic laboratories to achieve, but are ultimately necessary to ensure high-quality diagnostic reporting from this novel and highly complex technology. Without a doubt, the clinical diagnostic setting, including in the cardiological field, will see a dramatic increase in sequencing applications in the future.

### 4.4. Genetic Testing

The answer to why certain individuals develop myocarditis upon infection with common pathogens such as enteroviruses, or progress towards chronic forms of heart disease such as DCM or DCMi after acute phases, while others do not, may lie in part in genetic predisposition. In the context of viral etiologies of heart disease, a CCR5 gene mutation was found to correlate with spontaneous enteroviral clearance and improved outcomes in DCM or DCMi patients [245]. Isolated twin studies or familial clustering of virus-associated myocarditis or cardiomyopathy have been reported, but definitive proof of genetic predisposition is still lacking [246,247,248]. Most somatic mutations associated with cardiomyopathies cause changes in sarcomeric and cytoskeletal proteins, but also components involved in electrical conductivity and immune signaling. A recent multi-center study described an increased incidence of arrhythmogenic right ventricular cardiomyopathy- or DCM-associated genetic variants in patients with myocarditis (8%) versus healthy controls (<1%), giving the first insight into the possibility of these mutations increasing the chance of suffering from acute myocarditis apart from development of chronic heart disease [249]. The possible role of genetic variants in myocarditis and their known association with development of chronic heart disease has been extensively discussed elsewhere [250,251]. It should be clearly noted that while genetic testing does not require EMBs (as whole blood is sufficient) to support a suspected diagnosis, it cannot under any circumstance replace a clinical diagnosis of myocarditis or chronic heart disease merely on the basis the presence of a pathogenic gene variant, as so stated by the European Society of Cardiology Council on cardiovascular genomics [252].

## 5. Therapeutic Approaches

### 5.1. Immunosuppressive Therapy

Myocardial inflammation or systemic autoimmunity that persists after viral elimination warrants immunosuppressive treatment in addition to the optimal heart failure medications and after viral genome exclusion [253]. The importance of viral testing prior to immunosuppressive treatment has been shown in a retrospective analysis by Frustaci et al. in which viral persistence was associated with detrimental outcomes in patients with myocarditis receiving immunosuppressive therapy [253]. Therefore, detection or exclusion of a viral infection and possibly transcriptional viral activity by EMBs is necessary to initiate an immunosuppressive treatment. The prospective, randomized, placebo-controlled TIMIC study investigated patients with chronic, virus-negative myocarditis [254]. Here, 85 patients were randomized to receive corticosteroids plus azathioprine vs. placebo therapy. The results confirmed the beneficial effect of immunosuppression on LVEF recovery in a high number (88%) of patients at six months compared to placebo. Another non-randomized study analyzed the long-term outcome of *n* = 114 patients with virus-negative chronic myocarditis after immunosuppression with prednisolone and azathioprine. At follow-up, the patients showed a significant improvement in LVEF compared to baseline after a six-month period (LVEF rising from 44.6 ± 17.3 to 51.8 ± 15.5%, *p* = 0.006). After up to ten years, patients continued to show significant improvements in LV function [255]. 

In EMBs of patients with acute myocarditis, interleukin (IL) 1-beta mRNA levels are increased and inflammasome activation correlates with clinical severity [256]. In chronic heart failure, circulating IL-1b levels correlate with disease onset, severity, and mortality. Given this dual role of IL-1b in intramyocardial inflammation and heart failure, IL-1 inhibition has remarkable therapeutic potential in virus-negative DCMi. In patients with systemic inflammatory diseases, Anakinra controlled severe cases of myocarditis [257]. The CANTOS trial gave a proof-of-concept in controlling chronic inflammation via IL-1b inhibition with canakinumab in patients with coronary artery disease [258]. Its potential still needs to be proven in myocarditis. Furthermore, IL-6 is critical for the development of inflammation-driven fibrosis, leading to a reduction in macrophages and fibrotic changes. The IL-6 inhibitor tocilizumab has been used to effectively treat fulminant myocarditis in a single case reports [259]. 

The T_H_17 cell response appears to be one of the keys to the progression of chronic damage, cardiac fibrosis, and loss of cardiac function in autoimmune processes [56]. A case report of a patient diagnosed with autoimmune myocarditis associated with psoriasis demonstrated successful use of the IL-17 inhibitor secukinumab in the treatment of autoimmune myocarditis [260]. Furthermore, a recent study of 100 consecutive myocarditis patients revealed that psoriasis involvement occurred in 5% of patients, and complete cure occurred with secukinumab therapy over a six-month period [261]. However, the potential and safety of anti-IL-17 therapy remains to be investigated in larger trials.

The multicenter, double-blind, placebo-controlled, randomized phase 3 RHAPSODY trial provided evidence of the potential efficacy and safety of rilonacept, an IL-1α and β inhibitor, in chronic recurrent pericarditis [262,263]. This agent may also be considered as a potential therapeutic option for post-viral inflammatory processes. However, the therapeutic benefit for patients with inflammatory myocardial disease must also be highlighted in controlled trials.

In immune checkpoint inhibitor (ICI)-related acute myocarditis, therapeutic approaches are based on a smaller case series. High dose corticosteroids are considered the first line therapy [264]. 

Idiopathic giant cell myocarditis (IGCM) is a form of rapidly progressing myocarditis. It is important to note that IGCM represents the most aggressive form of acute myocarditis, often leading to a fatal outcome [200]. Therefore, treatment of IGCM should be initiated promptly. Anti-thymocyte globulin associated with high dose corticosteroids is preferred, together with cyclosporine starting a few days later. Azathioprine can be added. There are consistent data suggesting that discontinuation of immunosuppressive treatment after one year may be followed by relapse [21].

Looking towards the future, miRNAs have not only great potential as biomarkers in cardiac diagnostics, but as basis for specific therapies as well. Both miRNA mimics, as well as inhibitors (antisense nucleotides, or anti-miRs) have completed phase II studies, such as the miR-29-3p mimic remlarsen for the treatment of keloid scars, or the anti-miR-122-5p miravirsen as an antiviral strategy against hepatitis C virus [265,266]. A promising candidate for the treatment of heart failure and reduced LVEF after myocardial infarction (MI) is a miR-132-3p inhibitor (CDR132L) currently being investigated in the phase II HF-REVERT trial. Pre-clinical and phase I results showed good tolerability and beneficial effects on fibrosis and heart function after MI [267,268]. With the vast number of studies and scientific reports on the role of miRNAs in heart disease, it is only a matter of time until miRNA-based therapies for those with an inflammatory and/or viral etiology enter the field. 

### 5.2. Antiviral Therapy

The strongest antiviral trial-based data are for EVs and AdVs. In a non-randomized study, EV- and AdV-positive patients were treated with interferon beta (IFN β) [123]. IFN β treatment demonstrated complete elimination of the EV and AdV genomes by follow-up EMBs. Viral elimination was associated with improvement in mean LV function and prolongation of survival. Subsequently, a phase 2 study—Betaferon in Chronic Viral Cardiomyopathy (BICC) trial—was initiated. Study participants with EV-positive or AdV-positive EMB-proven myocarditis showed viral clearance after treatment with IFNβ. This was associated with favorable effects on NYHA functional class improvement and in quality of life [106]. In the same trial, IFNβ therapy was not associated with clearance of viral DNA in patients with B19V-positive myocarditis. In the BICC study, however, no distinction was made between latent B19V infection and the transcriptional activity of the virus, as transcriptional activity was not investigated at that time. Consequently, a sub-collective of B19V mono-infected patients was retrospectively analyzed. Interestingly, a positive effect of IFN β on B19V transcriptionally active patients was found, suppressing B19V viral RNA and improving hemodynamic outcome six months after treatment [105]. Further antiviral strategies against B19V infections are under investigation. For example, clinical improvement and reduction of transcriptional activity has been observed after antiviral therapy with the nucleoside analogue tenovofir [109] and telbivudine treatment [108]. Intravenous immunoglobulin (IVIg) therapy has been used in single case reports of patients with severe B19V viraemia. IVIg did not clinically improve B19V-associated chronic DCM, but transcriptional activity was not assessed [269]. In addition, IVIg therapy showed evidence of decreased mortality and LVEF improvement in children with acute myocarditis; however, its efficacy at improving outcomes remains controversial in adults [270]. Therefore, large randomized trials with long-term follow-up are needed to evaluate the efficacy of antiviral therapy in myocarditis and DCMi.

Treatment with acyclovir, ganciclovir, or valganciclovir may be considered for systemic herpesvirus infections, although their efficacy has not been directly studied in patients with myocarditis. Although elimination of a chromosomally integrated HHV-6 virus with persistently high viral load is not possible, transcriptional activity in iciHHV6 can be affected by treatment with valganciclovir [98]. To date, however, there have been no randomized studies on this. A single report of successful use of the anti-malarial drug artesunate in the treatment of a pediatric myocarditis case exists [100]. Artesunate has been shown to be more effective against HHV-6 than ganciclovir or valganciclovir, at least in cases of viral neurological disease [271]. Ganciclovir has been shown to be successful in individual reports of CMV- and EBV-associated myocarditis, but so far, there are no randomized studies on this either [87,92]. 

**Figure 4:**
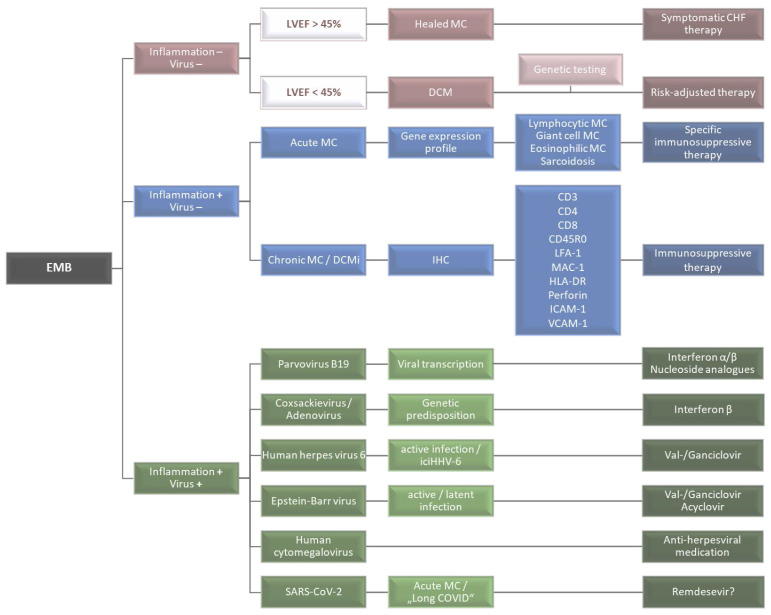
Schematic representation of the decision-making process on the basis of a comprehensive, differentiated, EMB-based diagnosis. Red: no evidence of inflammation and virus genomes; blue: detection of inflammatory infiltrates without detection of viral genomes; green: simultaneous detection of inflammation and viral genomes.

Finally, although not specifically indicated, the nucleoside analogues remdesivir or a combination of lopinavir and ritonavir have been used in certain cases of SARS-CoV-2 myocarditis [116]. However, canonical supportive and heart failure therapy remains more common. An overview of a comprehensive EMB diagnostics-based decision-making process for specific and causal therapy is depicted in Figure 4.

## 6. Conclusions

The diagnosis and effective treatment of myocarditis and DCMi remain a major clinical challenge. Despite the development of new imaging techniques, EMBs remain the gold standard in the accurate diagnosis of inflammatory myocardial disease as cardiac imaging techniques are not able to make a correct diagnosis based on pathophysiological mechanisms. With the introduction and on-going development of immunohistochemistry-based inflammation diagnostics and viral nucleic acid tests, EMB diagnostic methods have improved significantly since their introduction. The detection and differentiation of inflammatory infiltrates on the one hand and proof of active viral processes on the other hand is a prerequisite for adequate and personalized therapy decision making. Despite great progress in the last 30 years, biopsy-based diagnostics still has room for improvement. Promising new technologies such as miRNA and gene expression profiling, quantification of specific immune cell markers, and the determination of viral activity by the detection of RNA transcripts, will improve EMB diagnostics immensely. 

Progress has also been made in the treatment of myocarditis and DCMi. For example, it is now known that immunosuppressive therapy is contraindicated in virus-positive patients and that the use of antiviral drugs improves the prognosis of patients with viral myocarditis. Nevertheless, EMB diagnostics still suffer from low recognition, especially outside of European countries, even though modern imaging technologies fail to prove viral etiologies or detect low-grade or focal inflammatory processes. To further enhance the acceptance and validity of the use of EMBs, continuing advances in the biopsy-based diagnostic methodology and the resulting precision therapeutic options must be made in the future. In conclusion, although EMB still has room for development and growth, it offers enormous potential for improved causal and personalized treatment of patients with inflammatory or viral myocardial diseases.

## Figures and Tables

**Table 1:** Most commonly detected cardiotropic viruses, their cell tropism within the myocardium, associated clinical manifestations, and known or suspected pathophysiological aspects, as well as treatment options. ACE2 = angiotensin-converting enzyme 2; COVID = coronavirus disease; DCM = dilated cardiomyopathy; dsDNA = double-stranded DNA; iciHHV-6 = inherited chromosomally integrated HHV-6; DCMi = inflammatory cardiomyopathy; IVIg = intravenous immunoglobulins; MC = myocarditis; ssDNA = single-stranded DNA; ssRNA = single-stranded RNA. * Reports of virus-induced or -associated myocarditis are discussed in section “Virus diagnostics”.

Virus	Classification	Cardiac Tropism	Clinical Manifestation *	Pathophysiological Aspects	Treatment
Adenoviruses	dsDNA	cardiomyocytes	acute MC	direct cardiomyocyte damage	interferon β [80]
Epstein–Barr virus	dsDNA	B cells; CD8^+^ T cells [81]; cardiomyocytes [82,83]; endothelial cells [83]	acute MC; DCM [75]; reduced allograft survival [84]	indirect vascular damage linked to viral replication [85]; indirect (immune-mediated) damage [86]; possible direct cardiomyocyte infection [82,83]	ganciclovir [87]
Cytomegalovirus	dsDNA	endothelial cells [88]; leukocytes [89]; smooth muscle cells [90]	acute MC; DCM [75]; reduced allograft survival [91]	indirect (immune-mediated) damage; development of vasculopathy in donor hearts [91]	ganciclovir [92]
Enteroviruses	ssRNA	cardiomyocytes	acute MC; chronic MC; autoimmune DCMi; DCM [75]	direct cardiomyocyte damage [93]; non-cytopathic viral persistence [94]	interferon β [80]
Human herpes virus 6	dsDNA	T cells; endothelial cells [95]	acute MC; coronary artery spasm [96]; DCM [75];	viral persistence [79]; cardiomyocyte damage secondary to endothelial dysfunction [97]; iciHHV-6 reactivation and HHV-6 superinfection [98,99]	artesunate [100]; valganciclovir [98]
Parvovirus B19	ssDNA	endothelial cells [101]	coronary artery spasm [96]; viral DCMi; autoimmune DCMi; DCM [75]; reduced allograft survival [84,102,103]	viral persistence [79]; cardiomyocyte damage secondary to endothelial dysfunction [97,101]; viral transcriptional reactivation-induced pathogenic cellular gene expression [104]	interferon β [105,106]; telbivudine [107,108]; tenofovir [109]; IVIg
SARS-CoV-2	ssRNA	ACE2- and TRMPSS2-expressing cardiomyocytes [110], endothelial cells and macrophages [12,111]	acute MC [112]; chronic MC [113,114]	direct cardiomyocyte damage [115]; endothelial dysfunction; “long COVID” autoimmune myocarditis [113,114]	remdesivir; lopinavir; ritonavir [116]

## Data Availability

The data presented in this study are available on reasonable request from the corresponding author.
